# Divergent regeneration‐competent cells adopt a common mechanism for callus initiation in angiosperms

**DOI:** 10.1002/reg2.82

**Published:** 2017-08-27

**Authors:** Bo Hu, Guifang Zhang, Wu Liu, Jianmin Shi, Hua Wang, Meifang Qi, Jiqin Li, Peng Qin, Ying Ruan, Hai Huang, Yijing Zhang, Lin Xu

**Affiliations:** ^1^ National Key Laboratory of Plant Molecular Genetics CAS Center for Excellence in Molecular Plant Sciences Institute of Plant Physiology and Ecology Shanghai Institutes for Biological Sciences Chinese Academy of Sciences 300 Fenglin Road Shanghai 200032 China; ^2^ Pre‐National Laboratory for Crop Germplasm Innovation and Resource Utilization Hunan Agricultural University Changsha Hunan 410128 China; ^3^ University of Chinese Academy of Sciences 19A Yuquan Road Changsha Beijing 100049 China; ^4^ College of Life and Environment Sciences Shanghai Normal University Shanghai 200234 China; ^5^ Department of Instrument Science and Engineering Shanghai Jiao Tong University 800 Dongchuan Road Shanghai 200240 China

**Keywords:** angiosperm, callus, plant regeneration, rice, WOX11, WOX5

## Abstract

In tissue culture, the formation of callus from detached explants is a key step in plant regeneration; however, the regenerative abilities in different species are variable. While nearly all parts of organs of the dicot *Arabidopsis thaliana* are ready for callus formation, mature regions of organs in monocot rice (*Oryza sativa*) and other cereals are extremely unresponsive to tissue culture. Whether there is a common molecular mechanism beyond these different regenerative phenomena is unclear. Here we show that the *Arabidopsis* and rice use different regeneration‐competent cells to initiate callus, whereas the cells all adopt *WUSCHEL‐RELATED HOMEOBOX 11* (*WOX11*) and *WOX5* during cell fate transition. Different from *Arabidopsis* which maintains regeneration‐competent cells in mature organs, rice exhausts those cells during organ maturation, resulting in regenerative inability in mature organs. Our study not only explains this old perplexity in agricultural biotechnology, but also provides common molecular markers for tissue culture of different angiosperm species.

## 1 SUMMARY STATEMENT

*WOX11*−*WOX5* is a conserved molecular pathway adopted by different types of regeneration‐competent cells for callus initiation in angiosperms, and depletion of these cells results in regenerative inability in cereals.

### 1.1 Significance

An amazing feature of plant cells is their plasticity, which endows plants with remarkable regeneration abilities (Ikeuchi, Ogawa, Iwase, & Sugimoto, 2016; Sugimoto, Gordon, & Meyerowitz, 2011; Vogel, 2005; Xu & Huang, 2014) and has been widely exploited in agricultural technologies (Sussex, 2008). Detached or wounded plant organs usually form a group of fast dividing cell mass, termed callus, in different regenerative systems (Ikeuchi, Sugimoto, & Iwase, 2013). In tissue culture, de novo organogenesis could occur on a type of callus which has a high pluripotency for adventitious root and shoot regeneration (Ikeuchi et al., 2013; Kareem et al., 2016; Sugimoto et al., 2011; Xu & Huang, 2014).

Studies of *Arabidopsis thaliana* suggested that the callus formation on callus‐inducing medium (CIM) in tissue culture follows the rooting developmental pathway (Atta et al., 2009; Che, Lall, & Howell, 2007; He, Chen, Huang, & Xu, 2012; Liu et al., 2014; Sugimoto, Jiao, & Meyerowitz, 2010). Two steps of cell fate transition occurred in callus formation. *Arabidopsis thaliana WUSCHEL‐RELATED HOMEOBOX 11* (*AtWOX11*) is activated in the first step of cell fate transition from regeneration‐competent cells to founder cells; and cell division occurs in the second step of cell fate transition from founder cells to callus cells which are marked by *AtWOX5* (Liu et al., 2014).

The application of plant regeneration in tissue culture has occurred for more than half a century; however, a key obstacle in this biotechnology is that the ability for callus initiation upon hormone induction is highly diverse in different species. For example, while almost all organs of *Arabidopsis*, the typical dicot plant, have the ability to produce callus during their whole life (He et al., 2012; Sugimoto et al., 2010), mature regions of organs in many monocot cereal species are extremely unresponsive to in vitro culture techniques (Bhojwani, Evans, & Cocking, 1977; Cutler, Saleem, & Wang, 1991), perplexing the agricultural applications of tissue culture in cereal species for a long time. Whether there is a common molecular discipline for callus formation of different species is unclear, and thus how to explain the cereal problem in tissue culture is so far unanswered. In this study, we revealed this common discipline at the molecular level in angiosperms and this might be a marker to monitor tissue culture in future agricultural technologies. In addition, we discuss the regenerative inability in mature organs of cereals.

## 2 RESULTS

### 2.1 Identification of regeneration‐competent cells for callus initiation in rice

**Figure 1:**
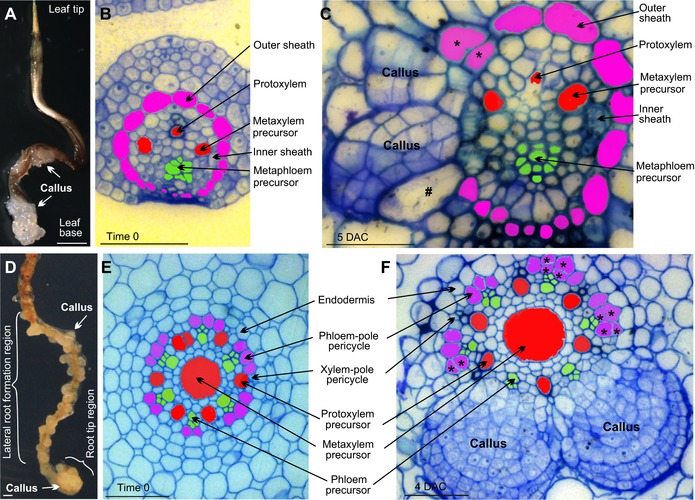
Cellular analysis of callus formation in rice. (A) Wild‐type rice leaf (7‐mm long) used as a source of explants for tissue culture on CIM for 2 weeks. (B), (C) Thin sections from time‐0 (B) and 5‐DAC (C) rice leaf explants cultured on CIM at leaf base. (C) Callus formed primarily from the outer sheath: *, the outer sheath cell that underwent division to form two callus cells; #, elongated outer sheath cell before cell division to form callus cells. Note that some inner sheath cells also underwent division. (D) Wild‐type rice root explants from 5‐day‐old seedling cultured on CIM for 2 weeks. Callus formed from the root tip region and the lateral root formation region. (E), (F) Thin sectioning of rice root explants cultured on CIM at time 0 (E) and 4 DAC (F). Note that callus formed from the phloem‐pole pericycle in (F); asterisks indicate phloem‐pole pericycle cells that underwent cell division to form two callus cells. Cell lineage in rice leaf and root tissue formation was described previously (Zeng et al., 2016). Scale bars: (A), (D) 1 mm; (B), (C), (E), (F) 50 μm

Dicots and monocots are two major branches of angiosperms, but the cellular and molecular mechanisms of regeneration in monocots are largely unclear. To analyze the cell lineage of callus formation in the monocot model plant rice (*Oryza sativa*), we cultured leaf and root explants on CIM. The mature rice leaf is unable to form callus in tissue culture (Bhojwani et al., 1977; Cutler et al., 1991). However, the base region of young leaves formed callus in our culture conditions (Fig. 1A) (Cutler et al., 1991), and this region is at the immature stage (Zeng et al., 2016). At the basal part of a young leaf explant about 7 mm in length, the vascular bundle was surrounded by two bundle sheath layers, i.e., the outer sheath and the inner sheath (Zeng et al., 2016) (Fig. 1B). Callus initiated primarily from the outer sheath at 5 days after culture (DAC) (Fig. 1C). Cell division could also be occasionally observed from the inner sheath (Fig. 1C). In the root, callus can be formed at the root tip region and the lateral root formation region (Fig. 1D). Callus initiated from the phloem‐pole pericycle (Fig. 1E, F), where lateral roots usually initiate during root development (Zeng et al., 2016). Therefore, bundle sheath cells in leaves and phloem‐pole pericycle cells in roots serve as regeneration‐competent cells for callus initiation in rice. It is possible that other immature cells in the vasculature may also be competent for callus initiation (see the analysis for maize, below).

### 2.2 *OsWOX11*/*12B* and *OsWOX5* are involved in callus formation in rice

To explore the molecular mechanism that confers the ability to regenerate on certain tissues in rice, we performed an RNA sequence experiment. We identified candidate genes that were highly upregulated during callus formation from the basal part of young leaf explants about 7 mm in length (Fig. S1; Table S1). *Oryza sativa WOX11* (*OsWOX11*) and *OsWOX12B*, which are intermediate‐clade *WOX* genes, and *OsWOX5* belonging to the WUS clade were among the highly upregulated genes (Fig. S1; Table S1) (van der Graaff, Laux, & Rensing, 2009; Haecker et al., 2004; Lian, Ding, Wang, Zhang, & Xu, 2014; Zeng et al., 2016).

**Figure 2:**
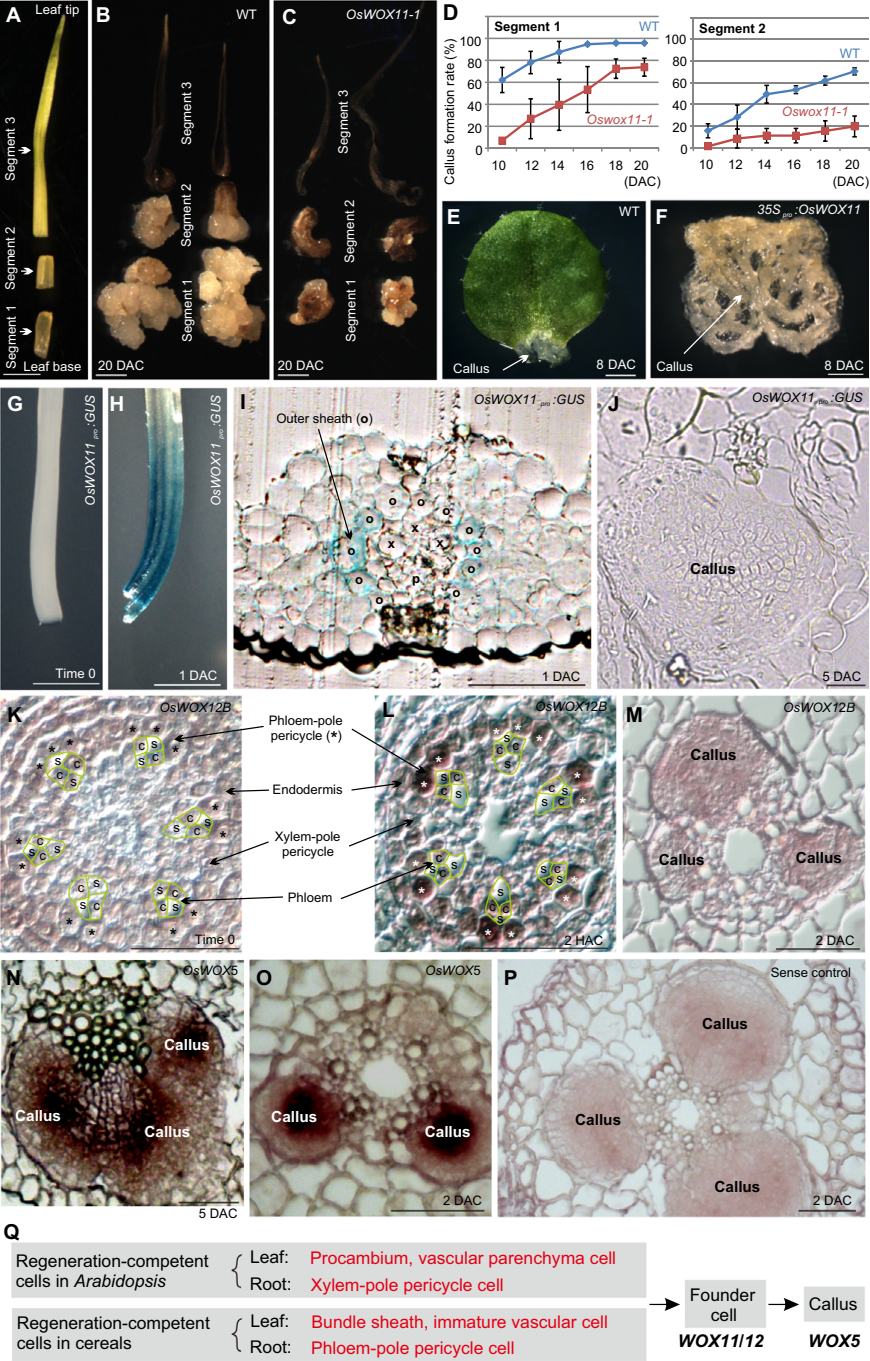
*OsWOX11*/*12B* and *OsWOX5* are involved in callus formation in rice. (A) Rice leaf (7 mm) as dissected at time 0 into three segments (1, 2, and 3). (B), (C) Dissected rice leaf explants (segments 1, 2, and 3) from wild type (Hwayoung) (B) and *Oswox11‐1* (C) cultured on CIM at 20 DAC. Note that the regenerative ability was weaker in *Oswox11‐1* than in wild type. (D) Callus formation rate analyses of cultured rice leaf segment explants. The rate was evaluated by counting the ratio of explants that formed callus. Bars show the SD of three biological repeats (*n* = 30 for each repeat). (E), (F) Callus formation in wild‐type *Arabidopsis* leaf explants (E) and *35S_pro_:OsWOX11 Arabidopsis* leaf explants (F) at 8 DAC. (G), (H) GUS staining at time 0 (G) and 1 DAC (H) in 7‐mm leaf explants from *OsWOX11_pro_:GUS* rice on CIM. (I), (J) Transverse section of leaf explants from *OsWOX11_pro_:GUS* rice on CIM at 1 DAC (I) and 5 DAC (J). Note that the GUS signal was present primarily in the outer sheath at 1 DAC (c), and occasionally could also be observed in some parenchyma cells and vascular cells: o, outer sheath; x, xylem; p, phloem. (K)–(M) Transverse sections at the tip region of the rice root explant at time 0 (K), 2 HAC (L), and 2 DAC (M), showing in situ hybridization of *OsWOX12B*. Note that *OsWOX12B* was not detected at time 0 (K), was located primarily in the phloem‐pole pericycle and occasionally in the endodermis at 2 HAC (L), and was absent from the callus at 2 DAC (M). The green lines indicate the four‐cell structure of phloem (Zeng et al., 2016); c, companion cell in phloem; s, sieve‐tube element in phloem; asterisks indicate the phloem‐pole pericycle. (N), (O) Transverse sections of rice leaf explants at 5 DAC (N) and rice root explants at 2 DAC (O) showing in situ hybridization of *OsWOX5* in callus. (P) Sense control. (Q) Model of cell fate transition during callus formation in *Arabidopsis* and cereals. Scale bars: (A)–(C), (E)**–**(H) 1 mm; (I)**–**(P) 50 μm

To test whether *OsWOX11* is involved in rice regeneration, we analyzed the callus formation ability of the rice *Oswox11‐1* mutant (Zhao, Hu, Dai, Huang, & Zhou, 2009). We dissected the 7‐mm young leaf explant into three segments: two 1‐mm segments (segments 1 and 2) at the leaf base and the remaining 5 mm as the third segment (segment 3) (Fig. 2A). In the wild type, callus formed in segments 1 and 2 but not in segment 3 (Fig. 2B). In the *Oswox11‐1* mutant, both segments 1 and 2 showed decreased callus formation rates compared with those of wild type (Fig. 2C, D). These data suggested that *OsWOX11* could be involved in callus formation in rice, and the partially reduced regenerative ability of the *Oswox11‐1* mutant may be due to the redundant function of *OsWOX12B*.

In addition, overexpression of *OsWOX11* in *Arabidopsis* resulted in dramatically rapid callus formation. In the *Arabidopsis* wild‐type Columbia‐0 (Col‐0) leaf explant, a small piece of callus was produced at the wounded region at 8 DAC (Fig. 2E), whereas callus formed everywhere on the *Arabidopsis* leaf explant from the *35S_pro_:OsWOX11* line (Fig. 2F).

Next, we analyzed the spatial expression pattern of *OsWOX* genes during rice callus formation. We constructed the transgenic rice line carrying *OsWOX11_pro_:GUS*. The GUS signal was not observed in time‐0 leaf explants (Fig. 2G), but was clearly present in the vasculature of leaf explants at 1 DAC (Figs. 2H and S2). A sectioning experiment showed that the GUS signal was primarily localized in the outer sheath at 1 DAC (Fig. 2I). The GUS signal disappeared when callus was undergoing rapid cell division at 5 DAC (Fig. 2J). The results of in situ hybridization analyses showed that, in the root tip region, the expression of *OsWOX12B* and *OsWOX11* was induced primarily in the phloem‐pole pericycle cells at 2 h after culture (HAC) and disappeared from the dividing callus at 2 DAC (Figs. 2K–M and S3). *OsWOX5* was detected in callus cells in both leaf and root explants (Fig. 2N–P). These spatial expression patterns indicated that the expression of *OsWOX11*/*12B* marks the appearance of founder cells for callus initiation while *OsWOX5* marks callus undergoing rapid cell division in rice. Therefore, *OsWOX11*/*12B* and *OsWOX5* may serve as molecular markers in cell fate transition during callus formation.

### 2.3 Angiosperms may have a common mechanism for callus initiation

The diversification of monocots and dicots was estimated to occur in the Jurassic (Zeng et al., 2014). In the dicot *Arabidopsis*, procambium and vascular parenchyma cells in leaves and xylem‐pole pericycle cells in roots serve as regeneration‐competent cells for callus initiation (Atta et al., 2009; Che et al., 2007; Liu et al., 2014; Yu et al., 2010). This suggests that regeneration‐competent cells differ between the dicot *Arabidopsis* and the monocot rice (see the model in Fig. 2Q). Whether the diverse regeneration‐competent cells in angiosperms share the same evolutionary lineage is not yet clear. However, the divergent regeneration‐competent cells act similarly at the molecular level during regeneration. *OsWOX11*/*12B* and their *Arabidopsis* homologs *AtWOX11*/*12* mark fate transition from regeneration‐competent cells to founder cells, and *OsWOX5* and its *Arabidopsis* homolog *AtWOX5* mark fate transition from founder cells to callus cells (Liu et al., 2014) (see the analysis of *Arabidopsis* callus formation in Fig. S4). In addition, WOX11 can activate *WOX5* expression in both *Arabidopsis* and rice (Hu & Xu, 2016) (see the activation of *OsWOX5* by OsWOX11 in Fig. S5).

To test whether this molecular pathway is generally involved in regeneration, we carried out quantitative reverse transcription polymerase chain reaction (qRT‐PCR) analyses to quantify the transcript levels of *WOX11* and *WOX5* homologs in the dicot poplar and the monocot maize during callus formation in leaf explants. Our data showed that expression levels of *WOX11* and *WOX5* homologs were dramatically induced on CIM in poplar and maize (Fig. S6). Therefore, it is possible that the molecular mechanism in regeneration could be conserved among angiosperms.

Overall, activation of *WOX5* is the marker of callus cell formation. We do not exclude the possibility that some non‐*WOX11*/*12*‐mediated pathways may also be able to activate *WOX5* for callus formation (Liu et al., 2014; Sheng et al., 2017).

### 2.4 Rice and *Arabidopsis* have different strategies for maintenance of regeneration‐competent cells during organ maturation

**Figure 3:**
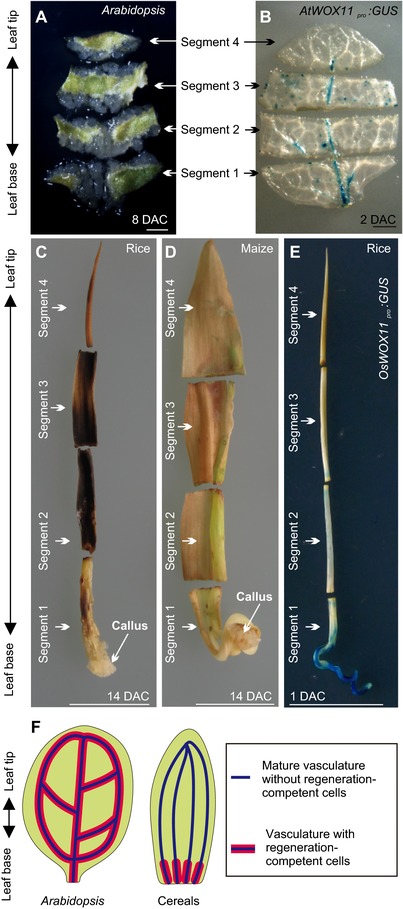
Different regenerative responses between the dicot *Arabidopsis* and monocot cereals. (A) Dissected mature *Arabidopsis* leaf explant cultured on CIM. All four segments were able to form callus. (B) GUS staining of mature leaf explants from *AtWOX11_pro_:GUS Arabidopsis* on CIM at 1 DAC. GUS signal was present in all four segments. (C), (D) Dissected rice leaf explant (5 cm) (C) and maize leaf explant (5 cm) (D) cultured on CIM, showing that only the very base segment (segment 1) was able to form callus. (E) GUS staining of 5‐cm leaf explants from *OsWOX11_pro_:GUS* rice on CIM at 1 DAC. Note that the GUS signal was strongly induced in leaf base segment (segment 1). (F) Model of regenerative abilities in leaves of *Arabidopsis* and cereals. Scale bars: (A), (B) 1 mm; (C)−(E) 1 cm

It is well known that the dicot *Arabidopsis* and monocot cereals have different regenerative abilities. Almost all organs of *Arabidopsis* are able to produce callus during the entire life of the plant (He et al., 2012; Sugimoto et al., 2010). We dissected mature *Arabidopsis* leaves into four segments (segments 1 to 4, from the base to the tip), and all dissected segments of leaf explants were able to form callus (Fig. 3A). In contrast, mature organs of many monocot cereal species are extremely unresponsive to in vitro culture techniques (Bhojwani et al., 1977; Cutler et al., 1991). We also dissected mature leaves from rice and maize into four segments from the base to the tip, and only the base region in segment 1 (immature region) was able to form callus on CIM (Figs. 3C, D and S7A). This is consistent with the results of previous studies showing that only the very base region of leaves from many monocot cereals, including barley, rice, wheat, oat, and maize, can form callus (Ahmadabadi, Ruf, & Bock, 2007; Becher, Haberland, & Koop, 1992; Chen, Xu, Loschke, Tomaska, & Rolfe, 1995; Chen, Zhuge, & Sundqvist, 1995; Cutler et al., 1991; Wernicke & Milkovits, 1984; Wernicke, Brettell, Wakizuka, & Potrykus, 1981; Zamora & Scott, 1983).

We hypothesize that the diversification of regenerative abilities in *Arabidopsis* and cereals is due to different strategies of maintenance of regeneration‐competent cells in mature organs. Regeneration‐competent cells, such as procambium cells, were maintained in *Arabidopsis* leaves as they matured. However, regeneration‐competent cells have differentiated into specific cell types (Zeng et al., 2016) and thus have lost their competence upon maturation from the tip region to the base region of the leaf in cereals. For example, the outer sheath in the rice mature leaf differentiated into large parenchyma cells (Zeng et al., 2016) (Fig. S7B). In maize, callus formation could be observed from many cells of the immature vasculature at the base region of the leaf explant when cultured on CIM (Figs. S7C, D and 2Q). During the development of the vasculature, callus formation could be observed primarily from the bundle sheath (Figs. S7C, E and 2Q). In mature maize leaves, the bundle sheath differentiated into Kranz anatomy (Fig. S7F) and therefore lost the ability to form callus.

To test this hypothesis at the molecular level, we analyzed expression patterns of *WOX11* in mature leaves of *Arabidopsis* and rice in tissue culture. We observed that *AtWOX11* was induced in the vasculature of all dissected segments of the mature *Arabidopsis* leaf on CIM (Fig. 3B); this may have rendered the whole *Arabidopsis* leaf competent to form callus at leaf maturity (see the model in Fig. 3F). In contrast, the GUS signal from the *OsWOX11_pro_:GUS* line was present at the base part of segment 1 (immature region), but was barely detected in segments 2−4 (mature region) of the dissected rice leaf explant on CIM (Fig. 3E). Therefore, as the rice leaves matured, the differentiation of regeneration‐competent cells resulted in the loss of their molecular competence for callus initiation (see the model in Fig. 3F).

Next, we tested the callus formation ability in rice leaf explants overexpressing *OsWOX11*. The data showed that overexpression of *OsWOX11* in rice cannot reverse the fate of mature and differentiated vascular cells to be competent for callus formation (Fig. S8). Callus formation requires not only *WOX11* but also many other molecular pathways such as *LATERAL ORGAN BOUNDARIES DOMAIN* genes (Fan, Xu, Xu, & Hu, 2012), *WOUND INDUCED DEDIFFERENTIATION 1* (Iwase et al., 2011; Iwase et al., 2017), and some epigenetic factors (He et al., 2012; Li et al., 2011) in *Arabidopsis*. *PLETHORA*s contribute to the pluripotency of callus (Kareem et al., 2015). It will be interesting to analyze these pathways during callus formation in rice and to test whether it is possible to endow differentiated cells with competence for callus formation in the future.

## 3 CONCLUSION AND PERSPECTIVE

In this study, we have provided cellular and molecular frameworks of callus formation in angiosperms. Regeneration‐competent cells differ between the dicot *Arabidopsis* and the monocot rice, whereas those diverse cells adopt a common mechanism involving *WOX11* and *WOX5* during cell fate transition for callus initiation. Depletion of regeneration‐competent cells during organ maturation may result in loss of regenerative ability in cereals.

Previous studies indicate that callus formation follows the rooting pathway (He et al., 2012; Liu et al., 2014; Sugimoto et al., 2010) and callus is a group of root primordium‐like cells (Liu et al., 2014). In addition, the regeneration‐competent cells for callus initiation could also initiate roots (Liu et al., 2014). During adventitious rooting in *Arabidopsis*, *AtWOX11* controls root founder cell establishment (Liu et al., 2014) and *AtWOX5* is required for root primordium formation (Hu & Xu, 2016). Based on these studies, we hypothesize that the *WOX11*−*WOX5*‐mediated root initiation mechanism in the common ancestor of angiosperms was borrowed and developed for callus initiation in regeneration‐competent cells of dicots and monocots, although the morphology of these cells has changed during evolution. Understanding of the regeneration‐competent cell behaviour in different plant species is the basis to utilize and improve the regenerative abilities in tissue culture.

## 4 MATERIALS AND METHODS

### 4.1 Plant materials

*Oryza sativa* L. japonica. cv. Nipponbare, *Arabidopsis thaliana* Col‐0, maize (*Zea mays*) B73, and poplar (*Populus davidiana* XP. *bollena* cv. Shan‐Xin) were used as wild types in this study unless otherwise noted. The *Oswox11‐1* mutant (PFG_2A‐00597, Hwayoung background) was described previously (Jeon et al., 2000; Jeong et al., 2006; Zhao et al., 2009).

To produce *OsWOX11_pro_:GUS* transgenic plants, the 4‐kb promoter of *OsWOX11* was PCR amplified and inserted into pBImUB (modified from pBI101). *AtWOX11_pro_:GUS* and *AtWOX5_pro_:GUS* transgenic plants were produced as described previously (Liu et al., 2014). For *OsWOX11* overexpression, cDNA fragments encoding the full‐length OsWOX11 protein were PCR amplified and inserted into pCAMBIA1300‐35S (modified from pCAMBIA1300) for overexpression in *Arabidopsis* or inserted into pCAMBIA1301‐UBiN for overexpression in rice. Transgenic plants were obtained by *Agrobacterium tumefaciens*‐mediated transformation into rice (Biorun, Wuhan, China) or *Arabidopsis*. The primers used for plasmid construction are listed in Table S2.

### 4.2 Tissue culture

Rice seeds were sterilized and placed on half‐strength Murashige and Skoog basal medium with 1% sucrose, 1% agar, and 0.5 g/L 2‐(*N*‐morpholino)ethanesulfonic acid (MES), pH 5.7 (Murashige & Skoog, 1962), for germination. For tissue cultures, sterilized explants were cultured at 29°C in darkness on CIM (N6 basal medium with 3% w/v sucrose, 0.3% w/v Phytagel, 0.5 g/L MES, pH 5.8, and 2,4‐dichlorophenoxyacetic acid) (Chu et al., 1975). The CIM was supplemented with 2,4‐dichlorophenoxyacetic acid at 2 mg/L for explants from rice, poplar, and maize. Tissue culture conditions for *Arabidopsis* were described previously (Liu et al., 2014).

### 4.3 Thin sectioning, *in situ* hybridization and dual luciferase assay

Thin sectioning was performed as previously described (Zeng et al., 2016). For in situ hybridization, gene fragments used to prepare probes were subcloned into pGEM‐T Easy. In situ hybridization analyses were performed as reported previously (Zeng et al., 2016). To construct *OsWOX5_pro_:LUC*, the promoter of *OsWOX5* was PCR amplified and inserted into the pGreenII‐0800 vector (Hellens et al., 2005). The dual luciferase assay was performed using the Dual‐Luciferase Reporter Assay System (Promega, Madison, WI). The primers used for plasmid construction are listed in Table S2.

### 4.4 qRT‐PCR and RNA‐sequencing analyses

RNA extraction and qRT‐PCR were performed as previously described (He et al., 2012), using gene‐specific primers. The qRT‐PCR results are shown as relative transcript levels, which were normalized against that of *ACTIN*. The primers used for real‐time PCR are listed in Table S2.

For RNA‐sequencing analyses, RNA was isolated from the base region of time‐0, 2‐DAC and 5‐DAC rice leaf explants (Fig. S1). Deep sequencing was carried out using the Illumina HiSeq3000 platform following the manufacturer's instructions (Illumina, San Diego, CA). Library construction and deep sequencing were performed by Genergy Biotechnology Co. Ltd (Shanghai, China). Raw data comprised 100‐bp paired‐end sequences. Raw sequences were aligned to the rice genome with TopHat software (Trapnell et al., 2012), and differential expressed gene analysis was performed using DESeq (Anders & Huber, 2010). Highly upregulated genes were defined as fold change >10.0 and *p* value <0.05. The analyzed data are shown in Table S1.

### 4.5 Accession numbers

The RNA‐sequencing data have been deposited in the Gene Expression Omnibus (http://www.ncbi.nlm.nih.gov/geo) under the accession number GSE86869. Sequence data can be obtained using the following accession numbers: Rice Genome Annotation Project, *OsWOX11* (LOC_Os07g48560), *OsWOX12B* (LOC_Os03g20910), and *OsWOX5* (LOC_Os01g63510); Genbank, *ZmWOX11A *(AM234774) and *ZmWOX5A* (AM234769); Phytozome, *PdPbWOX11* (Potri.013G066900) and *PdPbWOX5* (Potri.008G065400); and Arabidopsis Genome Initiative, *AtWOX11* (At3g03660) and *AtWOX5* (At3g11260).

## Supporting information


**Figure S1**. Identification of *OsWOX11*, *OsWOX12B*, and *OsWOX5* in rice callus formation.
**Figure S2**. *OsWOX11* expression during callus formation from leaf explant.
**Figure S3**. *OsWOX11* expression during callus formation from root explant.
**Figure S4**. *AtWOX11* and *AtWOX5* in *Arabidopsis* callus formation.
**Figure S5**. OsWOX11 activates *OsWOX5*.
**Figure S6**. *WOX11* and *WOX5* expression during callus formation in poplar and maize.
**Figure S7**. Leaf explants of rice and maize in tissue culture.
**Figure S8**. Overexpression of *OsWOX11* in rice.

**Table S1**. RNA‐seq data.
**Table S2**. List of primers used in this study.
