# Supplementary material for: Divergent regeneration‐competent cells adopt a common mechanism for callus initiation in angiosperms
Source: Regeneration (Oxf). 2017 Aug 27;4(3):132–9. doi: 10.1002/reg2.82 (PMC5617900; doi:10.1002/reg2.82)
Supplement: Supplementary file 1 — Figure S1. Identification of OsWOX11, OsWOX12B, and OsWOX5 in rice callus formation. Figure S2. OsWOX11 expression during callus formation from leaf explant. Figure S3. OsWOX11 expression during callus formation from root explant. Figure S4. AtWOX11 and AtWOX5 in Arabidopsis callus formation. Figure S5. OsWOX11 activates OsWOX5. Figure S6. WOX11 and WOX5 expression during callus formation in poplar and maize. Figure S7. Leaf explants of rice and maize in tissue culture. Figure S8. Overexpression of OsWOX11 in rice. [file REG2-4-132-s001.pdf]

Supporting Information

Divergent regeneration-competent cells adopt a common mechanism for callus initiation in angiosperms

Bo Hu[1,2,*,§], Guifang Zhang[1,3,*], Wu Liu[1,*], Jianmin Shi[1,4,*], Hua Wang[1,*], Meifang Qi[1,3], Jiqin Li[1], Peng Qin[5], Ying Ruan[2], Hai Huang[1], Yijing Zhang[1] and Lin Xu[1,3,#]

1, National Key Laboratory of Plant Molecular Genetics, CAS Center for Excellence in Molecular Plant Sciences, Institute of Plant Physiology and Ecology, Shanghai Institutes for Biological Sciences, Chinese Academy of Sciences, 300 Fenglin Road, Shanghai 200032, China.

2, Pre-National Laboratory for Crop Germplasm Innovation and Resource Utilization, Hunan Agricultural University, Changsha, Hunan 410128, China

3, University of Chinese Academy of Sciences, 19A Yuquan Road, Beijing, 100049, China

4, College of Life and Environment Sciences, Shanghai Normal University, Shanghai 200234, China

5, Department of Instrument Science and Engineering, Shanghai Jiao Tong University, 800 Dongchuan Road, Shanghai 200240, China

§, Present address: Department of General Genetics, Center for Molecular Biology of Plants (ZMBP), University of Tübingen, Auf der Morgenstelle 32, 72076 Tübingen, Germany

Running Title: Callus formation in rice

* These authors contributed equally to this work.

Author for correspondence
E-mail: xulin01@sibs.ac.cn

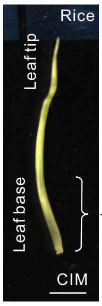

→ RNA-seq

2 DAC vs. Time 0

Gene	Locus identifier	Fold change	*p* value
OsWOX12B	LOC_Os03g20910	1027.38	4.16E-03
OsWOX11	LOC_Os07g48560	749.79	5.97E-04

5 DAC vs. Time 0

Gene	Locus identifier	Fold change	*p* value
OsWOX12B	LOC_Os03g20910	829.23	2.62E-03
OsWOX11	LOC_Os07g48560	293.82	9.84E-04
OsWOX5	LOC_Os01g63510	115.55	4.60E-03

Figure S1. Identification of *OsWOX11*, *OsWOX12B* and *OsWOX5* in rice callus formation. RNA-seq analysis of gene transcripts was performed using the base region (~2 mm) of 7-mm wild-type rice leaf explants at time 0, 2 DAC and 5 DAC on CIM. *OsWOX11* and *OsWOX12B* were identified as highly upregulated genes from time 0 to 2 DAC. *OsWOX11*, *OsWOX12B* and *OsWOX5* were identified as highly upregulated genes from time 0 to 5 DAC. Also see RNA-seq data in Table S1.

Scale bar, 1 mm.

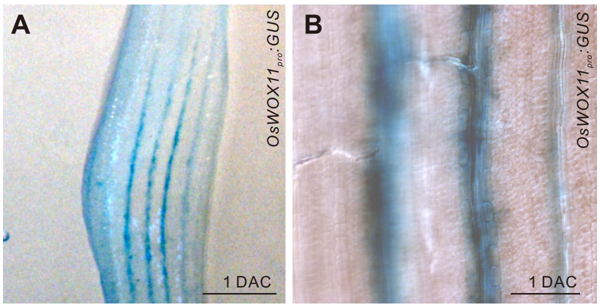

Figure S2. *OsWOX11* expression during callus formation from leaf explant. (A, B) GUS staining of the 7-mm leaf explants from $OsWOX11_{pro}$:*GUS* rice at 1 DAC on CIM. Note that *OsWOX11* was induced in leaf vasculature at the base region. (B) is the close-up view of (A).

Scale bars, 1 mm in (A) and 100 μm in (B).

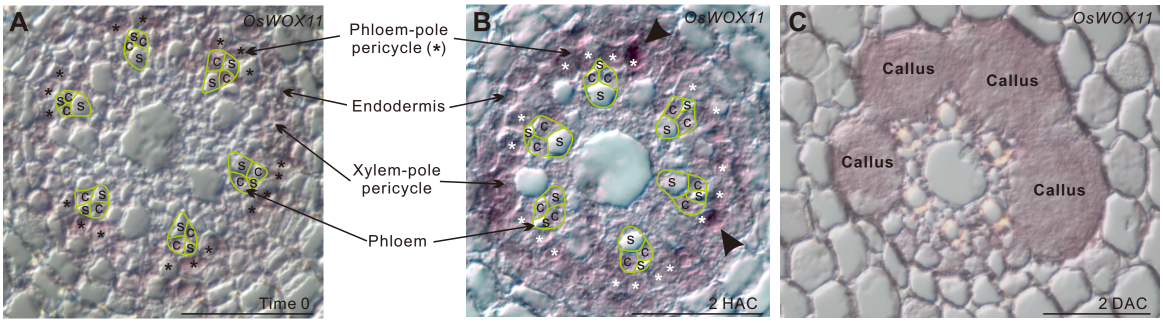

Figure S3. *OsWOX11* expression during callus formation from root explant.

(A–C) Transverse sections of rice root tip explant at time 0 (A), 2 HAC (B), and 2 DAC (C), showing *in situ* hybridization of *OsWOX11*. Note that *OsWOX11* is induced in phloem-pole pericycle cells (arrowheads) at 2 HAC. Green lines indicate phloem. c, companion cell in phloem; s, sieve-tube element in phloem. Asterisks indicate phloem-pole pericycle.

Scale bars, 50 μm in (A–C).

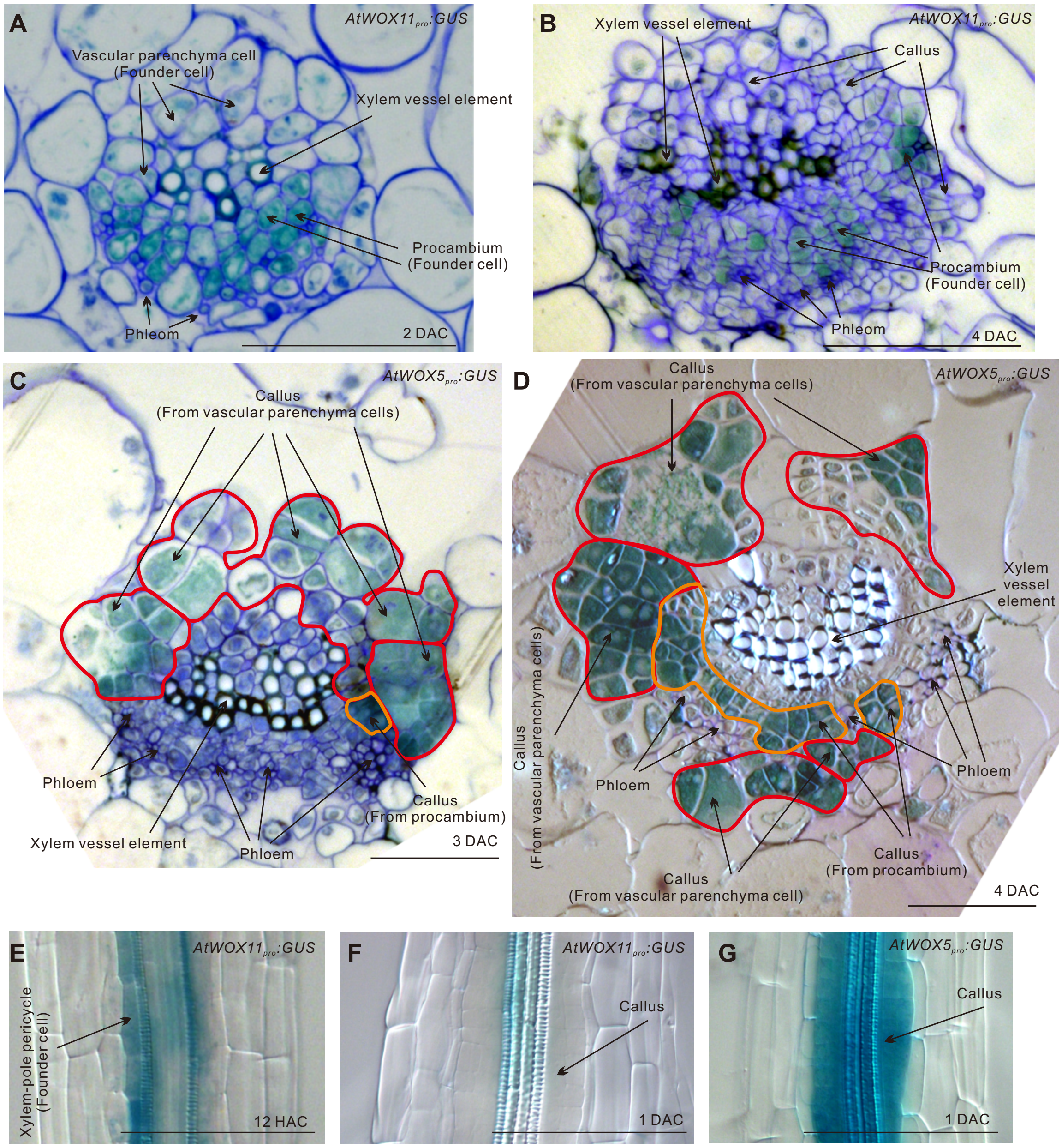

Figure S4. *AtWOX11* and *AtWOX5* in *Arabidopsis* callus formation.

(A, B) Transverse sections of *Arabidopsis* leaf explants from $AtWOX11_{pro}$:*GUS* at 2 DAC (A) and 4 DAC (B) on CIM. Note that expression of *AtWOX11* could be highly induced in procambium and vascular parenchyma cells at 2 DAC (A), and its expression decreased at 4 DAC in callus cells (B). Therefore, *AtWOX11* marked fate transition from regeneration-competent cells (procambium and vascular parenchyma cells) to founder cells.

(C, D) Transverse section of *Arabidopsis* leaf explants from $AtWOX5_{pro}$:*GUS* at 3 DAC (C) and 4 DAC (D) on CIM. Note that *AtWOX5* was highly expressed in the newly formed callus cells, and those fast dividing cells were derived from procambium (indicated by orange lines) and vascular parenchyma cells (indicated by red lines).

(E, F) *Arabidopsis* root explants from $AtWOX11_{pro}$:*GUS* at 12 HAC (E) and 1 DAC (F) on CIM. *AtWOX11* is induced in xylem-pole pericycle cells at 12 HAC, which serve as regeneration-competent cells (E). Note that *AtWOX11* could occasionally be observed in some other vascular cells (E). *AtWOX11* expression reduced at 1 DAC when callus was under fast cell division (F).

(G) *Arabidopsis* root explants from $AtWOX5_{pro}$:*GUS* at 1 DAC on CIM. *AtWOX5* was expressed in callus cells.

Scale bars, 100 μm in (A–G).

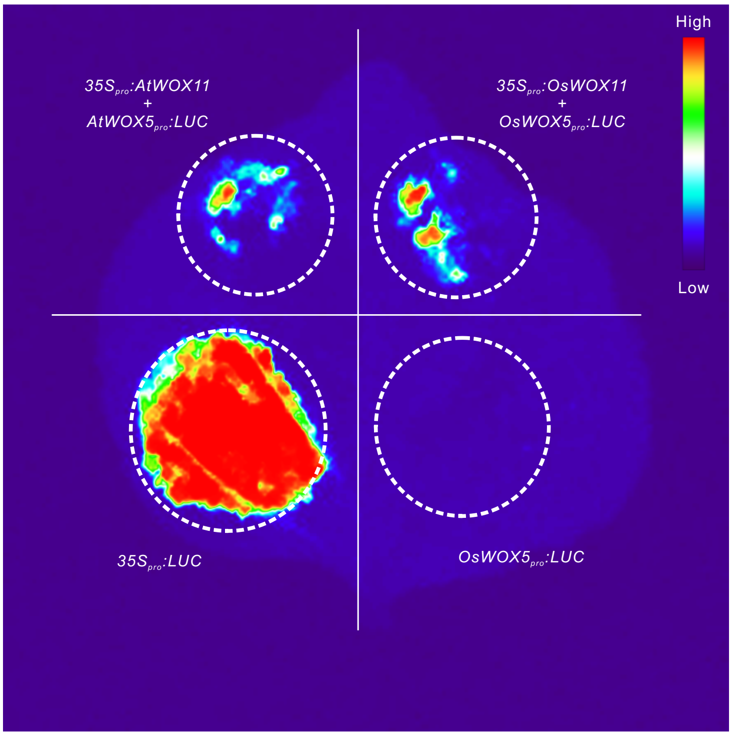

Figure S5. OsWOX11 activates *OsWOX5*.

Coexpression of *35S*$_{pro}$:*OsWOX11* together with *OsWOX5*$_{pro}$:*LUC*, in which the luciferase reporter gene is fused downstream of the *OsWOX5* promoter, in a transient expression system in tobacco leaves. Note that OsWOX11 activated *OsWOX5*$_{pro}$:*LUC* luciferase response *in planta*. The *35S*$_{pro}$:*LUC* construct and the *35S*$_{pro}$:*AtWOX11*/*AtWOX5*$_{pro}$:*LUC* pair (Hu & Xu 2016) served as positive controls. *OsWOX5*$_{pro}$:*LUC* served as a negative control.

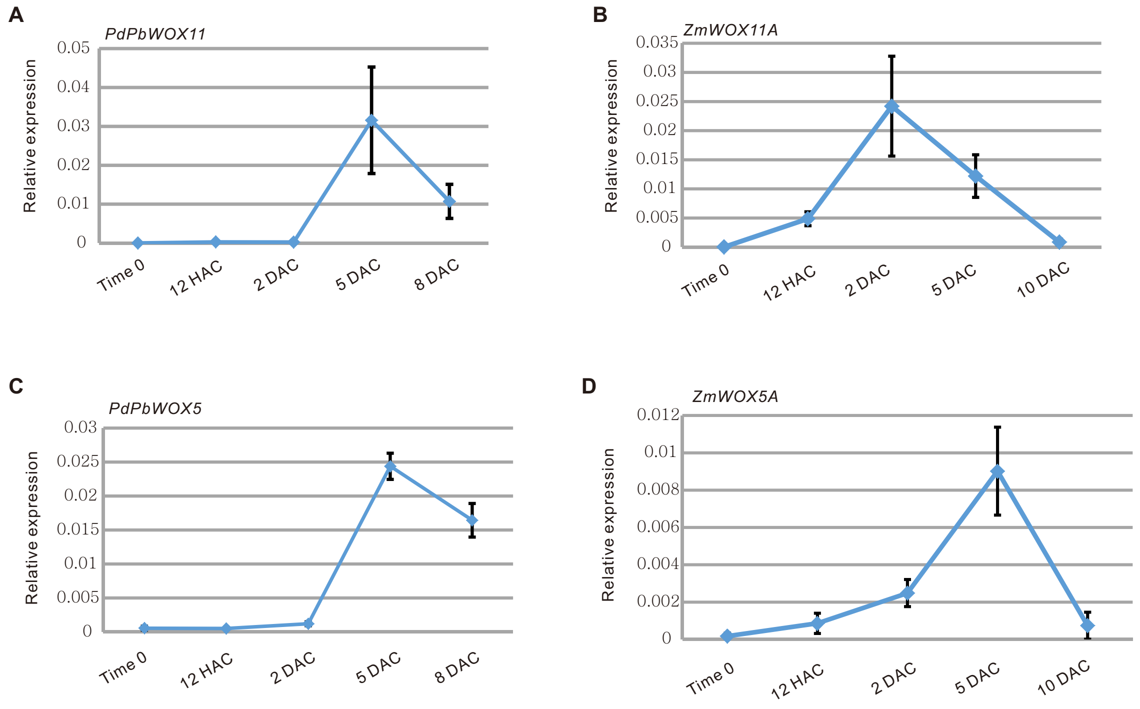

Figure S6. *WOX11* and *WOX5* expression during callus formation in poplar and maize.

(A, B) Expression of poplar *PdPbWOX11* (A) and maize *ZmWOX11A* (B) in leaf explants cultured on CIM.

(C, D) Expression of poplar *PdPbWOX5* (C) and maize *ZmWOX5A* (D) in leaf explants cultured on CIM. Whole leaf explants were used for poplar, and base region from a 7-mm leaf was used for maize tissue culture. Bars show SEM from three biological repetitions. Each biological repetition was performed with three technical repetitions.

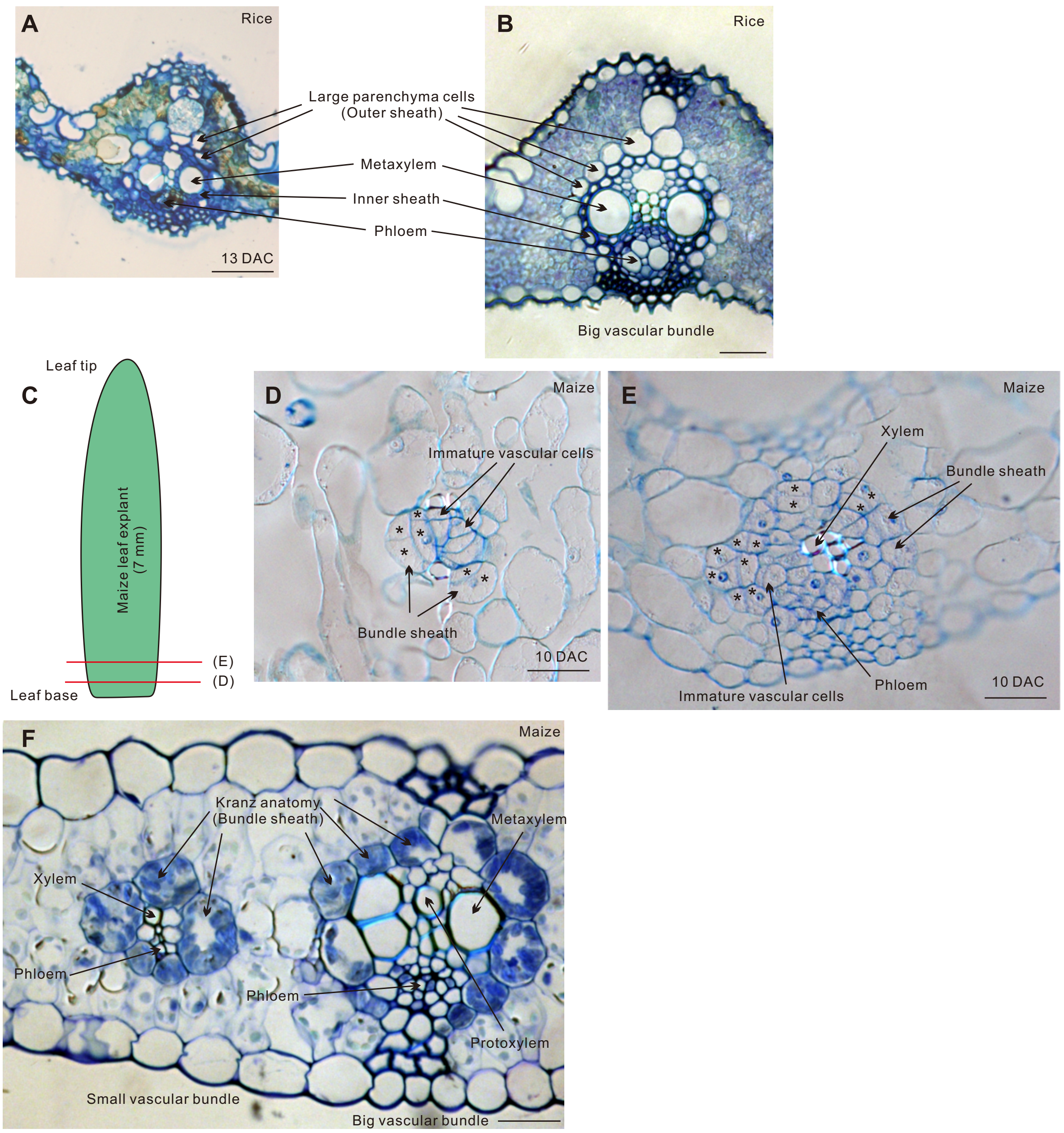

Figure S7. Leaf explants of rice and maize in tissue culture.

(A) Thin section from the mature rice leaf cultured on CIM, showing unresponsiveness to CIM.

(B) Thin section from a mature rice leaf. Note that the outer sheath differentiated into large parenchyma cells (Zeng et al. 2016)

(C) Diagram of sectioning positions in (D) and (E).

(D, E) Thin sections from different positions of a 7-mm maize leaf explant at the base region at 10 DAC on CIM. The vasculature was in the immature stage in (D) and cell division could be observed in many vascular cells. The vasculature was partially differentiated in (E), and callus was initiated primarily from the bundle sheath and occasionally observed from some immature vascular cells. Asterisks indicate the bundle sheath cells that underwent cell division.

(F) Thin section from a mature maize leaf. Note that bundle sheath differentiated into Kranz anatomy.

Scale bars, 50 μm in (A, B, D–F)

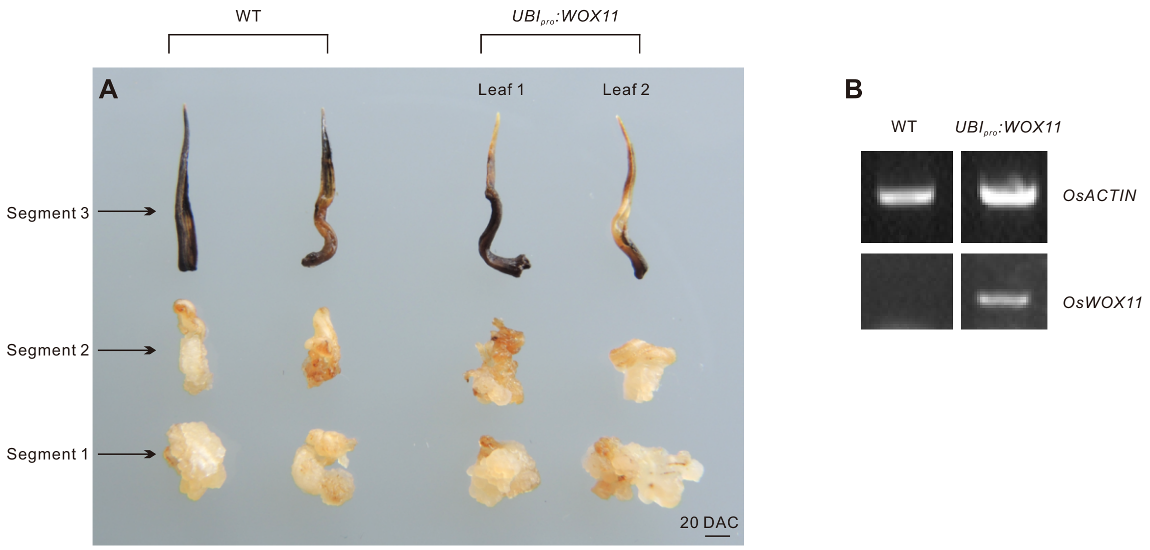

Figure S8. Overexpression of *OsWOX11* in rice.

(A) Rice leaf explants from wild type and *UBI*$_{pro}$:*OsWOX11* cultured on CIM at 20 DAC.

We dissected 7-mm young leaf explants into three segments as described in Fig. 2A.

Callus formed in segments 1 and 2 but not in segment 3 in both wild type and *UBI*$_{pro}$:*OsWOX11*.

At least 30 leaves were tested from two transgenic lines and two leaves from one transgenic line were shown (leaf 1 and leaf 2 from line 8).

(B) RT-PCR analysis of *OsWOX11* expression in rice leaves of wild type and *UBI*$_{pro}$:*OsWOX11* (line 8).

Scale bar, 1 mm in (A).

Table S2. List of primers used in this study.

Experiments	Primers	Sequence (5'→3')
Molecular cloning		
OsWOX11$_{pro}$:GUS	pOsWOX11-F1	acgcgtcgacTATGGCACTGCATGTCACATCTTG
	pOsWOX11-R1	cgggatcccGCCACTAGCTAGCTGCCTTGTTCG
35S$_{pro}$:OsWOX11 in *Arabidopsis*	OsWOX11-F1	cgcggatccATGGACGGCGGCCACAGCCCGGAC
	OsWOX11-R1	acgcgtcgacTCGCTCAACTCGATCAagacg
UBI$_{pro}$:OsWOX11 in rice	OsWOX11-F2	caccATGGACGGCGGCCACAGCCC
	OsWOX11-R2	AGACGACCTCGTGACCAGGA
OsWOX5:LUC	pOsWOX5-F1	acgcgtcgacGCCGGTCAGCGTACATCCATTGC
	pOsWOX5-R1	cgggatccGACCGACCGACTGATCGATCACT
OsWOX5 probe	OsWOX5-F1	ATGGAGGCTCTTAGCGGGCGAG
	OsWOX5-R1	ACTAGGACTAGGCACAGCGACA
OsWOX12B probe	OsWOX12B-F1	CGACGATCACGGTGTTCATC
	OsWOX12B-R1	GAGAAGAGACGCGACCATATTG
OsWOX11 probe	OsWOX11-F2	GAGTTGAGCGATTCGTCGATTG
	OsWOX11-R2	AGATCGAGAACGGGATACATAC
qRT-PCR		
PdPbWOX11	PdPbWOX11-F1	CTGGTTGCAGGAGAGCAAAC
	PdPbWOX11-R1	GACGGGGATTGAACAAAAGAAGG
PdPbWOX5	PdPbWOX5-F1	ATGGAAGAGAGAATGTCAGGC
	PdPbWOX5-F1	CGTTCTTGCTCTCGATCTTG
PdPbACTIN	PtPbACTIN-F1	GCGATTCCGTTGCCCA
	PtPbACTIN-R1	GGATGCCTGCAGCTTCCAT
ZmWOX11A	ZmWOX11-F1	GAGCAGATACTCATCCTCGAG
	ZmWOX11-R1	ACCAGTAGAAGACGTTGGCG
ZmWOX5A	ZmWOX5-F1	GAGCAGGTGA AGGTCCTGAC
	ZmWOX5-R1	TGGTTCTGGAACCAGTAGAAG
ZmACTIN	ZmACTIN-F1	GCTACGAGATGCCTGATGGTC
	ZmACTIN-R1	CCCCCACTGAGGACAACG
OsWOX11	OsWOX11-F1	CTACTACTCGTGTCAACCTG
	OsWOX11-R1	GGAAGTAGCTCTCGCCCATC
OsACTIN	OsACTIN-F1	GGTATTGTTAGCAACTGGGATG
	OsACTIN-R1	GATGAAAGAGGGCTGGAAGA

Note that lower case letters represent additional nucleotides to introduce restriction sites or Gateway cloning sequence.

References

Hu, X., Xu, L. 2016. Transcription factors WOX11/12 directly activate *WOX5/7* to promote root primordia initiation and organogenesis. Plant Physiol. **172**(4):2363-2373.

Zeng, M., Hu, B., Li, J., Zhang, G., Ruan, Y., Huang, H., et al. 2016. Stem cell lineage in body layer specialization and vascular patterning of rice root and leaf. Sci Bull. **61**(11):847-858.
